# Recent Advances in Molecular and Genetic Research on Uveal Melanoma

**DOI:** 10.3390/cells13121023

**Published:** 2024-06-12

**Authors:** Aurélie Fuentes-Rodriguez, Andrew Mitchell, Sylvain L. Guérin, Solange Landreville

**Affiliations:** 1 Department of Ophthalmology and Otorhinolaryngology-Cervico-Facial Surgery, Faculty of Medicine, Université Laval, Quebec City, QC G1V 0A6, Canada; aurelie.fuentes-rodriguez.1@ulaval.ca (A.F.-R.); andrew.mitchell@crchudequebec.ulaval.ca (A.M.); sylvain.guerin@fmed.ulaval.ca (S.L.G.); 2 Hôpital du Saint-Sacrement, Regenerative Medicine Division, CHU de Québec-Université Laval Research Centre, Quebec City, QC G1S 4L8, Canada; 3 Centre de Recherche en Organogénèse Expérimentale de l‘Université Laval/LOEX, Quebec City, QC G1J 1Z4, Canada; 4 Université Laval Cancer Research Center, Quebec City, QC G1R 3S3, Canada

**Keywords:** uveal melanoma, liquid biopsies, novel biomarkers, molecular mechanisms, emerging therapeutics, challenges

## Abstract

Uveal melanoma (UM), a distinct subtype of melanoma, presents unique challenges in its clinical management due to its complex molecular landscape and tendency for liver metastasis. This review highlights recent advancements in understanding the molecular pathogenesis, genetic alterations, and immune microenvironment of UM, with a focus on pivotal genes, such as *GNAQ*/*11*, *BAP1*, and *CYSLTR2*, and delves into the distinctive genetic and chromosomal classifications of UM, emphasizing the role of mutations and chromosomal rearrangements in disease progression and metastatic risk. Novel diagnostic biomarkers, including circulating tumor cells, DNA and extracellular vesicles, are discussed, offering potential non-invasive approaches for early detection and monitoring. It also explores emerging prognostic markers and their implications for patient stratification and personalized treatment strategies. Therapeutic approaches, including histone deacetylase inhibitors, MAPK pathway inhibitors, and emerging trends and concepts like CAR T-cell therapy, are evaluated for their efficacy in UM treatment. This review identifies challenges in UM research, such as the limited treatment options for metastatic UM and the need for improved prognostic tools, and suggests future directions, including the discovery of novel therapeutic targets, immunotherapeutic strategies, and advanced drug delivery systems. The review concludes by emphasizing the importance of continued research and innovation in addressing the unique challenges of UM to improve patient outcomes and develop more effective treatment strategies.

## 1. Introduction

Uveal melanoma (UM) is the most common primary intraocular malignancy in adults, accounting for approximately 3.8% of all melanomas (predominantly in the Caucasian population) [1], and whose development may be influenced by genetic ancestry [2]. Although rare, its prognosis is often poor, with a high propensity for liver metastasis and limited effective therapeutic options (reviewed in [3,4,5]). Predominantly originating from the choroid, and less frequently from the iris and ciliary body, UM presents a clinical profile distinct from other forms of melanoma, particularly in its patterns of metastasis and response to therapies. It exhibits distinct genetic, cellular, and molecular profiles, making it a unique entity (reviewed in [6,7]). Advances in molecular biology and genomics have uncovered a unique molecular landscape that includes specific mutations and chromosomal alterations, such as mutations in *GNAQ*/*11* (G protein subunits alpha q/11), *BAP1* (BRCA1-associated protein 1), *CYSLTR2* (cysteinyl-leukotriene receptor 2), and *PLCβ4* (phospholipase C beta 4), offering insights into the pathogenesis and potential therapeutic targets (reviewed in [3]). These molecular discoveries have led to the subclassification of UMs which, in turn, has significant prognostic implications and has become integral to patient management, guiding therapeutic decisions and enabling personalized treatment approaches (reviewed in [8]). Signaling pathways play a pivotal role in tumorigenesis and progression in many cancers, such as the JAK/STAT, mTOR and β-catenin pathways, among others (reviewed in [9,10,11,12,13]). Understanding these pathways and the broader immune microenvironment offers new targets for therapeutic interventions [14]. Simultaneously, emerging therapeutic strategies, including immunotherapies and targeted treatments, are providing new avenues for personalized care and improved outcomes [15,16,17]. However, despite these advancements, the management of UM, especially metastatic UM (MUM), remains a challenge. This review aims to provide a comprehensive overview of the current state of knowledge in UM, encompassing its molecular pathogenesis, diagnostic and prognostic biomarkers, current treatment modalities, and emerging therapeutic strategies. Additionally, we address the ongoing challenges in the field and propose future directions for research and clinical management, with the goal of improving outcomes for patients with this complex and aggressive cancer.

## 2. Genetic Landscape of UM: Latest Findings

**Figure 1:**
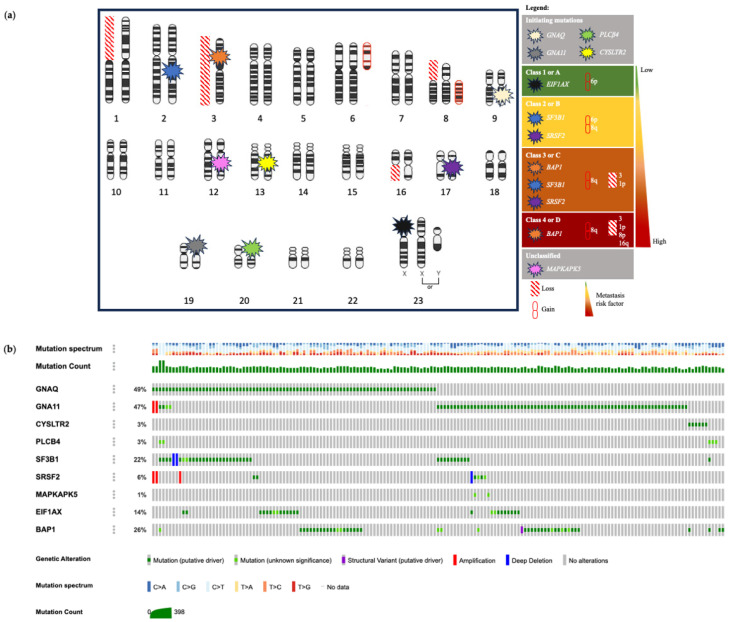
Comprehensive cytogenetic landscape: chromosomal aberrations, genetic classifications, and mutation profiles in UM. (**a**) Detailed classification of chromosomal and genetic alterations associated with UM. Key genes are identified by their respective location on the chromosomes and categorized based on their roles in the disease progression defined by indicators of chromosomal losses (red diagonal stripes) or gains (red circular icon) and mutations (starburst symbols in various colors). Initiating mutations (mutually exclusive) in genes like *GNAQ*, *GNA11*, *PLCβ4* and *CYSLTR2* are often the first genetic changes in UM development. The *EIF1AX* mutation and 6p gain are marked as Class 1 alterations, typically indicating a less aggressive form of UM. *SF3B1*/*SRSF2* mutations are found in Class 2 with chromosomal gains in 6p and 8q, or in Class 3 with monosomy 3 and 1p/8q gains, suggesting an intermediate prognosis. *BAP1* mutations are split between Class 3 with loss of chromosomes 3 and 1p or gain of chromosome 8q, and Class 4 with losses of chromosomes 3, 1p, 8p or 16q and gain of chromosome 8q, usually indicating a more severe prognosis due to their association with higher metastatic risk. The unclassified mutation *MAPKAPK5*, while not traditionally linked to a specific risk class, is included to underscore the genetic diversity of the disease. Even if cytogenetic, GEP and molecular genetic prognosticators are important to collect since they can allow a more accurate prognostication, they are not included yet in clinical staging algorithms, such as the 8th edition of the AJCC staging system for UM [88]. The AJCC anatomic staging continues to be essential when genetic prognostication is unavailable or not offered to patients. (**b**) The cBioPortal (an open-source platform that provides visualization, analysis, and downloading of large-scale cancer genomics data sets; https://www.cbioportal.org/ accessed on 8 June 2024) provides a detailed overview of primary UM mutations across the *GNAQ*/*11*, *CYSLTR2*, *PLCB4*, *SF3B1SRSF2*, *MAPKAPK5*, *EIF1AX*, and *BAP1* genes within the TCGA [14] and QIMR [49] UM cohort (*n* = 188 individuals). The percentage of mutations found in each gene is indicated on the left side of the visualization. Each vertical line in the visualization corresponds to individual patient data, where gene alterations, mutation spectrum and mutation count are mapped.

UM is defined by a unique landscape characterized by various genetic, cellular, and molecular alterations that collectively drive the development and progression of this eye cancer. Factors influencing the risk of UM include having fair skin, light-colored eyes, exposure to ultraviolet radiation, and specific inherited genetic mutations [18,19,20,21,22]. Iris, ciliary body and choroidal melanomas are the three uveal types that present unique clinical and genetic characteristics (reviewed in [7]). A comparative understanding of the UM originating from these three distinct ocular structures is essential for tailored therapeutic strategies and patient management. In contrast to cutaneous melanomas (CMs), which possess a higher tumor mutational burden, the majority of UMs originate from the choroid, a sun-protected ocular tissue, resulting in a lower mutational frequency [23,24,25]. The primary driving forces in UM are early mutations in genes such as *GNAQ*/*11*, *CYSLTR2* and *PLCβ4* (so-called initiating mutations), followed by later mutations (so-called prognostic mutations, excluding *MAPKAPK5*) in genes such as *SF3B1* (splicing factor 3B subunit 1), *SRSF2* (serine- and arginine-rich splicing factor 2), *MAPKAPK5* (MAPK activated protein kinase 5) and *EIF1AX* (eukaryotic translation initiation factor 1A X-linked), along with inactivating mutations in *BAP1*, a known tumor suppressor linked to a high risk of developing metastases when mutated (reviewed in [26]). Unlike CM, activating mutations in *BRAF* (B-Raf serine/threonine kinase) or *NRAS* (N-Ras GTPase) are rare or even absent in UM [27,28]. However, some approaches identified the T1799A point mutation in *BRAF* in posterior UM [29]. The clinical management of UM benefits significantly from stratification of patients into specific prognostic groups. This stratification informs treatment decisions and guides patient enrollment in clinical trials. Current prognostic tools analyze various factors, including tumor size, location, gene expression profile (GEP), mutations and chromosomal rearrangements [30,31,32]. For example, UMs can be divided into three distinct subtypes according to the expression of twelve discriminating mRNA transcripts (DecisionDx-UM GEP test): class 1A (2% 5-year metastatic risk; Low risk = Low intensity management with image-based surveillance every 12 months), class 1B (21% 5-year metastatic risk; Intermediate risk = Moderate intensity management with image-based surveillance every 6–12 months), and class 2 (72% 5-year metastatic risk), known for its aggressive nature and frequent progression to fatal metastatic disease (High risk = High intensity management with image-based surveillance every 3–6 months and discussion for preventive treatment or clinical trial opportunities) [33,34]. Over time, UM cases have been subclassified into four molecularly distinct and clinically relevant subtypes (1–4 or A–D) [14,35]. The better-prognostic subgroups are Class 1 or A (with disomy 3, *EIF1AX* mutation and 6p gain) and Class 2 or B (with disomy 3, *SF3B1*/*SRSF2* mutations and gains in 6p/8q), which are both associated with a moderate risk of developing MUM at a later stage. Conversely, the poor-prognostic subgroups are Class 3 or C (characterized by monosomy 3, *SF3B1*/*SRSF2*/*BAP1* mutations, and 8q gain) and Class 4 or D (with monosomy 3, *BAP1* mutations, 8q gain and/or multiple chromosomal copies) (reviewed in [36]) [14,35]. These classifications based on transcriptomic signatures, DNA methylation profiles, mutations and/or chromosomal alterations have proven to be prognostically significant, shaping personalized prognosis and treatment approaches in UM care. This section delves into recent scientific advancements concerning pivotal genes and their associated proteins, notably *GNAQ*/*11*, *BAP1*, *CYSLTR2*, *PLCβ4* genes, and additional research outcomes, which significantly impact the pathophysiology and prognostic factors of UM (Figure 1).

### 2.1. GNAQ/11 Mutations

*GNAQ/11* genes, encoding GTP binding proteins, are integral to activating the protein kinase C (PKC) enzyme and transmitting signals to the mitogen-activated protein kinase (MAPK) pathway. Studies have revealed activating mutations in *GNAQ* and *GNA11*, in codons Q209 or R183 (located in the ras-like domain) [37,38], in 85% to 94% of UM cases across all disease stages (reviewed in [39,40]) [41]. These early-event mutations are detected even in benign uveal nevi and are mutually exclusive [37,38]. They promote tumor growth and survival by persistently activating cell proliferation pathways, notably MAPK/ERK, leading to uncontrolled cell division and tumor progression [42]. A recent discovery includes the *GNAQ* hotspot mutation in codon G48 (located in the phosphate-binding loop) [43]. Active Gαq structural studies show that G48, R183, and Q209 mutations are close to the nucleotide-binding pocket [44]. Therefore, mutations in G48, as with Q209 and R183, could impair GTPase activity in similar ways. Further complexities in *GNAQ/11* functions have been unveiled, including the existence of multiple active states of G proteins [45]. Additionally, UM patients with heterogeneous *GNAQ*/*11* mutations in their tumor detected by droplet digital PCR had a higher likelihood of poor prognosis compared to those with none or homogeneous mutations, underscoring the influence of genetic heterogeneity on outcomes [46].

### 2.2. CYSLTR2 Mutation

*CYSLTR2*, a G-protein-coupled receptor, has been implicated in the development of 2–4% of UM cases [14,35,47]. The newly discovered specific mutation in codon L129 is considered an initial oncogenic event in tumors with wild-type *GNAQ* and *GNA11* genes [48]. The mutant allele abundance increased with tumor progression, while an increase in the wild-type allele frequency has been observed in UM tumors with mutations in *GNAQ*, *GNA11* or *PLCB4*, indicating a complex relationship between these genetic alterations [48].

### 2.3. PLCβ4 Mutation

PLCβ4, an enzyme pivotal in cellular signaling, plays a role in the hydrolysis of phosphatidylinositol 4,5-bisphosphate (PIP2) into second messengers, diacylglycerol (DAG), and inositol 1,4,5-trisphosphate (IP3). These messengers are key in activating protein kinase C (PKC) and releasing calcium from intracellular stores [14,35]. In UM, a mutation in *PLCβ4*, specifically at codon D630, is identified in a small fraction of cases (2.5%), and is classified as an initiating mutation [49] that constitutively activates the PLCβ/ε, PKCδ/ε, and MAPK signaling pathways [42,50]. When PKC isoforms δ and ε are activated, they induce the Ras-guanine nucleotide exchange factor RasGRP3, which triggers downstream pathways, such as the MEK/ERK axis that is important in UM tumorigenesis [51,52]. However, inhibitors of the PKC/MEK/ERK axis are rarely effective in the clinic (reviewed in [3]), suggesting that PLCβ4 promotes tumorigenesis through an alternate pathway. Indeed, a recent study performed in the *Tg(mitfa:PLCB4^D630Y^);tp53^M214K/M214K^;mitfa^−/−^* zebrafish line showed an active, nuclear-localized YAP1 (yes-associated protein 1) but a lack of phosphorylated ERK indicative of PLCβ signaling in these melanomic tumors [53].

### 2.4. SF3B1 Mutations

*SF3B1* somatic missense mutations appear in 15–29% of UM cases [54], and are present in prognostic subgroups Classes 2/B or 3/C. They occur mostly at the 625 arginine residue (R625) with other rare spots, such as lysine 666 (K666) [55,56]. The *SF3B1* gene encodes the subunit 1 of the splicing factor 3b protein complex, which is essential in pre-mRNA splicing to create canonical spliced transcripts; however, when mutated, the spliceosome complex uses alternative recognition sites resulting in aberrant spliced transcripts [57]. UM patients (n = 143 participants) who have *SF3B1* mutations exhibited metastatic disease both early and late in their diagnosis, categorized as occurring before or after a follow-up period of 60 months [58]. Earlier research established that mutations in *BAP1* and *SF3B1* are mutually exclusive in UM cases [14]. However, simultaneous presence of *BAP1* deficiency and *SF3B1* mutation in UM cells results in senescence due to an impaired DNA damage response [59]. This suggests a potential synthetic lethal interaction dependent on the genetic and epigenetic context [59]. Furthermore, mutations in *SF3B1* in UM tumors lead to changes in splicing that produce tumor neoepitopes restricted to MHC class I, which are recognized by the patient’s CD8^+^ T-cells [60]. Additionally, neoepitopes derived from *SF3B1*-independent alternative splicing isoforms AMZ2P1 and MZT2B have been identified as good potential antitumor candidates, since the production of IFN-γ and UM cell death were increased when incubated with CD8^+^ T-cells [61]. Emerging evidence indicates that alternative splicing dysregulation is a common feature of cancers that can have important clinical implications in diagnosis, prognosis and therapies [62]. For example, short exons are more sensitive to be dysregulated regardless of the cancer types, and a cancer-associated short exon-based panel was a strong pan-cancer predictor for survival [62].

### 2.5. SRSF2 Mutations

SRSF2 is also a protein part of the spliceosome which is involved in extending transcription and maintaining genomic integrity, therefore contributing to both the structural organization and the regulation of alternative splicing processes in precursor mRNA [63]. Mutation analysis of *SRSF2* in UM tumors revealed only few patients (4–6%) with in-frame deletions at different protein residues (p.(Tyr92_His99del); p.(Gly93_His100del); p.(Ser174_Ser179del)) [14,64,65,66], and they are found in prognostic subgroups Classes 2/B or 3/C. These mutations enhance the binding affinity of the mutant SRSF2 protein for the CCNG nucleotide sequence compared to the GGNG sequence, leading to changes in the rates of exon inclusion [65]. An overall downregulation of cancer hallmark genes was found when splicing factors such as *SRSF2* and *SF3B1* were mutated in UM, unlike other kinds of cancers [65]. It is recognized that the dysregulation of alternative splicing can increased tumor heterogeneity, cellular plasticity and altered metabolism, which will impact the therapeutic response (reviewed in [67]).

### 2.6. EIF1AX Mutations

Located on the X chromosome, the *EIF1AX* gene encodes for eukaryotic translation initiation factor 1A, a key component in the formation of the 43S pre-initiation complexes vital for protein synthesis [68,69]. In UM, mutations in *EIF1AX* are frequently observed (14–20% of all cases; prognostic subgroup 1/A), positioning it as an UM oncogenic factor. Mutant variants in exons 1 and 2 have been reported to enhance overall protein synthesis [49], aligning with the increased demand for protein synthesis typically seen in cancer cells (reviewed in [70]). 

### 2.7. BAP1 Mutations

The *BAP1* gene, located on chromosome 3, harbors loss-of-function mutations associated with various cancers, including UM (found in poor-prognostic subgroups Classes 3/C or 4/D) (reviewed in [71]). In conjunction with ASXL1/2/3 (additional sex combs like 1/2/3), it constitutes the polycomb repressive deubiquitinase complex. This complex primarily acts to detach monoubiquitin from H2AK119ub1 (ubiquitinated histone 2A at lysine 119) [72]. In more than 40% of UM cases, BAP1 loss leads to a stem-like state, affecting melanocyte differentiation and possibly driving metastasis [14,73,74,75]. The BAP1 protein exhibits an intricate structure with extensive intrinsically disordered protein regions [76], facilitating complex interactions and possibly influencing disease pathways. A recent epigenetic discovery revealed a negative correlation between BAP1 expression and cg01493712 DNA methylation [77], adding to the complexity of understanding BAP1 function.

### 2.8. MAPKAPK5 Mutations

MAPKAPK5 is a serine/threonine protein kinase, also referred to as p38-regulated and activated kinase (PRAK) or MK5, which is activated via the canonical MAPK pathway. It initiates and controls a variety of cellular functions, including proliferation, differentiation, apoptosis, and gene expression (reviewed in [78]). It is found mutated in around 2% of UM cases [14]. The TCGA analysis identifies two primary alterations at residues Q473Nfs* (frameshift mutation of the glutamine in position 473 leading to the insertion of a premature stop codon) and E106Kfs*23 (frameshift mutation of the glutamic acid in position 106 replaced by a lysine, leading to a sequence of 23 altered amino acids before encountering a premature stop codon); however, the effects of these mutations have not been thoroughly investigated in UM.

### 2.9. Chromosomal Alterations

Chromosomal alterations are recognized as essential indicators of prognosis and risk stratification in UM (Figure 1), and the proportion of the genome modified by copy number alterations (CNAs) differed significantly among patients, ranging from 0 to 53% [79]. Among these, chromosomes 3, 8p and 1p losses serve as independent predictors of distant metastasis [80]. More specifically, monosomy 3 and larger tumor size are frequently observed in high-risk UM patients, contributing to our understanding of the genetic factors that may lead to a more aggressive disease course [81]. Recent advances in the study of low-frequency CNAs have revealed an ultra-high-risk group characterized by monosomy 3 (44.7%), 8q amplification (41.8–47.5%) and deletion of 1p or 16q (15%), offering a more nuanced understanding of the underlying genetic landscape [79]. Additionally, a retrospective case-control study has shed light on specific chromosomal abnormalities in melanoma located in the anterior uvea, such as monosomy 3p, trisomy 6p and trisomy 8q, further diversifying our knowledge of the genomic complexity and heterogeneity in different UM subtypes [82].

### 2.10. Latest Findings

Recent research has identified polymorphisms in *BARD1* (breast cancer 1 (BRCA1) associated RING domain 1; rs1048108, rs2229571 and rs2070094) and *BRIP1* (BRCA1 interacting protein helicase 1; rs4986764) genes in patients with UM and progressive choroidal nevus (i.e., small melanocytic neoplasm with signs of growth within 2 years of observation) [83]. These findings are currently being explored to assess risk groups, prevention and diagnosis of UM and intraocular neoplasms [83]. In addition to these findings, a particular case study has further emphasized the genetic complexity of UM, revealing the presence of multiple mutations, including a mutation in the *PBRM1* (polybromo 1) gene coding for the BAF180 (BRG1-associated factor 180) protein involved in chromatin remodeling [84]. Lastly, *LRP1B* (low density lipoprotein receptor-related protein 1B) and *CHEK2* (checkpoint kinase 2) genes have been found mutated in UM samples and may be associated with high-risk phenotypes [85,86,87]. This adds to the understanding of the heterogeneous and multifaceted nature of this disease.

Advancements in the molecular understanding of UM are paving the way for more precise diagnostic, prognostic, and therapeutic approaches. The recognition of specific mutations, chromosomal alterations, and anatomical distinctions within UM subtypes provides an evolving landscape for more personalized medicine. Early events in mutations of *GNAQ*/*11* have been expanded with insights into active states, and further complexity has been recognized in the structure and regulation of BAP1. Recent attention to *CYSLTR2* mutations and advancements in understanding chromosomal alterations have enriched our understanding of the genetics of UM. The nuanced variations in tumor localization, specifically between ciliary body and choroidal melanomas, have also been highlighted, offering insights into targeted patient management and treatment strategies.

## 3. Novel Biomarkers

In recent years, significant advancements have been made in identifying novel biomarkers for UM diagnosis and prognosis, which has transformed patient care. In particular, there is increased interest in liquid biopsies, a non-invasive technique that allows researchers to extract vital tumor information from various bodily fluids, such as the analysis of circulating tumor cells (CTCs), circulating tumor DNA (ctDNA) and extracellular vesicles (EVs) (reviewed in [89,90,91,92]). New RNA biomarkers, specific genes, and immune indices have been explored for precise UM classification, metastasis prediction and prognosis assessment. These advancements have the potential to revolutionize UM diagnosis, allowing for more personalized and less intrusive care, therefore contributing to the refinement of predictive models, and facilitating targeted therapeutic interventions. Research into immune subtypes, innovative computational techniques, and the application of single-cell technologies also promise to improve diagnosis, prognosis, and personalized treatment plans for UM. The following sections present the latest findings on novel biomarkers for analysis, prognosis and personalized treatment.

### 3.1. Novel Biomarkers for Diagnosis

**Figure 2:**
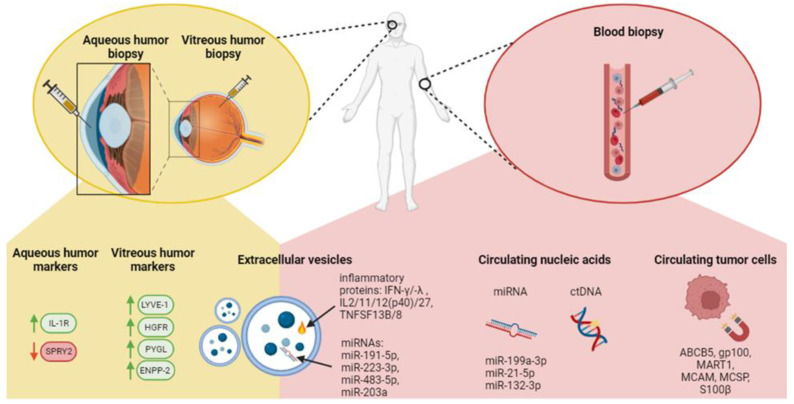
Liquid biopsy in UM diagnosis. A schematic representation of non-invasive liquid biopsy approaches for personalized patient care in UM. Key diagnostic biomarkers are depicted for each method (from the right to the left): Aqueous and vitreous humor biopsies provide an alternative non-invasive diagnostic approach to tumoral tissue-based GEP, revealing tumor markers such as SPRY2 and IL-1R in aqueous humor, and LYVE-1, HGFR, PYGL and ENPP-2 upregulation in vitreous humor. Extracellular vesicles: Elevated levels in UM patients’ plasma, containing disease-specific miRNAs that serve as diagnostic markers. Circulating nucleic acids: Identification of miRNA levels that are associated with UM, and utility of ctDNA analysis for the detection of UM-specific gene mutations, offering insights into tumor genetics and dynamics without a tumoral tissue biopsy. Circulating tumor cells: Isolated using UM-specific markers coupled to magnetic beads, enabling detection and analysis of tumor cells traveling in the bloodstream.

The analysis of components, such as CTCs, ctDNA, and EVs, from sources like blood, aqueous humor and vitreous humor, has the potential to profoundly transform UM diagnosis and clinical management. Unlike traditional tissue biopsies, which can be more intrusive, the liquid biopsy offers a less invasive means of accessing valuable insights (Figure 2).

#### 3.1.1. Circulating Tumor Cells (CTCs)

CTCs are shed into the bloodstream from primary tumors or metastases and could hold significant potential in UM as a clinical tool since they allow early cancer detection, provide a non-invasive method for diagnosis and monitoring, can help assess treatment response and can offer insights into the genetic makeup of the malignancy (e.g., prognostic or druggable mutations) (reviewed in [93,94]). CTC screening requires three steps, namely their capture, their identification and downstream analyses. Even now, the existence of numerous platforms using various technologies to detect the tumor cells (e.g., label-dependent or label-free detection) impact the reproducibility and applicability of CTC screening in daily clinical practice. A recent study comparing four platforms to capture UM cells in blood or culture medium, including the immunomagnetic CellSearch platform that was approved by the FDA in the early 2000s for the diagnosis and prognosis of metastatic cancers, failed to recover enough UM cells for further comparisons or molecular analyses [95]. Capturing and counting CTCs have traditionally been difficult because methods often target just a single surface antigen. However, a recent study involving a small group of UM patients (*n* = 43) demonstrated a significant improvement, detecting CTCs in 86% of UM patients from an 8 mL blood sample [96]. This success was achieved using a UM-specific bead approach that utilizes multiple markers (e.g., ABCB5, gp100, MART1, MCAM, MCSP, S100β) [96]. Lately, research has focused on a new group of CTCs known as circulating hybrid cells (CHCs) [97,98]. These CHCs possess features of both tumor cells and macrophages identifiable through their combined expression of tumor-related (gp100^+^, HTR2B^+^) or leukocyte-specific proteins (CD45^+^) [99]. In the context of UM metastatic progression (*n* = 68 UM patients), it was found that 92% of UM patients with >8 CHCs/50,000 nuclei cohort, experienced progression to metastatic disease within 3 years following their initial treatment [99]. Although CTC screening using multiple markers is promising, the biggest challenge for its use in routine clinical practice will be to establish an international consensus among the ocular oncology centers.

#### 3.1.2. Circulating Tumor DNA (ctDNA)

Cell-free DNA (cfDNA) is present in the bloodstream, originating from normal cellular activity or cell death. Its level naturally increases in response to various conditions, including stroke, autoimmune diseases, trauma, heart attack or cancer (reviewed in [100]). In the context of cancer, ctDNA is specifically released by tumor cells due to apoptosis, necrosis or active secretion [101]. In addition to CTCs, ctDNA can play a diagnostic role by detecting CNAs or UM gene mutations. Nevertheless, there is considerable variability in the literature regarding ctDNA detection in primary UM, with detectability rates ranging from 2 to 100% depending on the technique used (reviewed in [94]). This inconsistency makes the use of ctDNA in diagnosing primary UM still a subject of debate and underscores the need for better detection methods. For example, a recent study using a CRISPR/Cas12a-based fluorescent sensor was able to detect the *GNAQ* Q209P mutation in patients’ plasma with a minimum of 68 ctDNA copies/mL and 3% of fractional abundance of mutant *GNAQ* [102]. Although this technology is highly sensitive, the plasma from only four patients was tested using this technique. Therefore, further investigation with a larger cohort is needed.

#### 3.1.3. Extracellular Vesicles (EVs)

Intercellular communication is a fundamental biological process where cells exchange information to coordinate functions. One such method of intercellular communication is through the secretion of EVs, which are membrane-bound particles released into the extracellular space [103]. Both serum and plasma are known to contain EVs that encapsulate DNA, RNA, miRNAs and proteins (reviewed in [94,103,104]). Interestingly, UM-specific EVs have been identified in conditioned culture media and biological fluids [105,106,107,108]. In fact, not only is the EV content increased in UM patients (*n* = 7), but 39% of their cargo is conserved across various liquid biopsy sources, such as aqueous humor, vitreous humor and plasma [109]. Therefore, if UM-specific markers could be identified, EVs could be useful biomarkers for detecting and monitoring UM. Although no such UM-specific EV marker has currently been established, UM-derived EVs have some unique characteristics. For example, serum-derived exosomes (<200 nm vesicles) from MUM patients (*n* = 20) have an upregulation of inflammation-related proteins compared to healthy controls. This includes ILs (interleukins 2, 11, 12p40, 27) in metastatic-derived exosomes, as well as IFN-γ and -λ (interferon-gamma and -lambda) [110]. In addition, the presence of TNFSF-13B and TNFSF-8 (tumor necrosis factor ligand superfamily members 13B and -8) were found enriched in metastatic UM exosomal cargos [110]. Finally, UM exosomes contained extracellular matrix modifying proteins, such as PTX3 (pentraxin-3), MMPs (matrix metalloproteinases 1, 2, 3), osteopontin and osteocalcin, in comparison to healthy exosomes [110]. Nevertheless, the diagnostic usefulness of EVs in UM remains unclear due to a scarcity of studies in the field. Future research is essential to explore their potential more comprehensively as diagnostic/prognostic tools or drug delivery vehicles.

#### 3.1.4. Aqueous and Vitreous Humor Markers

Aqueous and vitreous humor biopsies have been proposed for UM patients with small tumors not eligible for a biopsy, or as a way to avoid potential risks associated with tumor biopsies, such as retinal detachment and the spread of the cancer [111]. This less invasive sampling could serve as UM-specific liquid biopsies for proteomics and real-time intraocular evaluation. Supporting this idea, research involving 20 UM patients found measurable levels of ctDNA in the aqueous humor (~0.1 mL) following brachytherapy treatment, with concentrations varying from 44.6 to 3,113 ng/mL [112]. Using the ctDNA, researchers also detected SCNAs (monosomy 3, 6p gain, 6q loss, and 8q gain) and UM-specific mutations (*GNAQ* and *BAP1*), suggesting the potential of this method for predicting outcomes and tracking the progression of UM post-radiation therapy [112]. Aqueous humor (*n* = 20 participants) also contains protein biomarkers correlating with the metastatic stage, such as SPRY2 downregulation and IL-1R upregulation [113]. A small sample study of UM patients (*n* = 36) was able to detect GNAQ, BAP1, SF3B1 and EIF1AX proteins in aqueous humor [114]. They provided data on expression levels in UM aqueous humor samples relative to a control group, but did not establish a link with clinical aspects, such as tumor thickness and basal diameter [114]. In addition, the vitreous humor of UM patients (*n* = 8) with high-risk GEP tumors showed higher expression of LYVE-1 (lymphatic vessel endothelial hyaluronan receptor 1), HGFR (hepatocyte growth factor receptor), PYGL (liver glycogen phosphorylase) and ENPP-2 (ectonucleotide pyrophosphatase/phosphodiesterase 2) proteins in comparison to controls (*n* = 3) [115]. The analysis of cytokines (*n* = 32) revealed that twenty-six were differentially expressed in the vitreous humor from UM patients compared to the control group [116]. Among these, five cytokines (PDGFAB/BB, G-CSF, MCP-3/CCL7, IL-13 and TNF-β) were found to be elevated in the group identified as high-risk of metastasis according to the GEP analysis [116].

#### 3.1.5. miRNA Biomarkers

Circulating microRNAs are small, single-stranded, non-coding RNA molecules that control gene expression post-transcriptionally by repressing translation or prompting degradation of specific target messenger RNAs. Since they are often found to be abnormally regulated in human cancers, including UM [117,118], miRNAs have shown potential as blood-based biomarkers for diagnosing cancer [119]. A recent study (*n* = 20 participants) demonstrated increased levels of miR-199a-3p, miR-21-5p and miR-132-3p in the serum of UM patients, with miR-199a-3p showing superior diagnostic effectiveness [120]. It was observed that the serum level of miR-199a-3p increased from the early stages through to the metastatic phase of UM [120]. The function of miR-199a-3p in UM and the detailed processes it influences require additional investigation. Finally, in MUM patients (*n* = 20), miRNAs hsa-miR-191-5p and hsa-miR-223-3p were found to be highly regulated in UM exosomes and might serve as potential biomarkers to detect early stages of UM [121].

### 3.2. Novel Biomarkers for Prognosis and Personalized Therapies

In the evolution of UM research, novel markers and gene signatures continue to refine our understanding of prognosis and therapeutic responses. PRAME (preferentially expressed antigen in melanoma), typically expressed in normal testis and exhibiting abnormal expression across many cancer types, has garnered significant attention in UM research due to its association with negative prognostic factors, where approximately 25% of UM tumors expressed PRAME in association with aneuploidy, metastasis and poor patient outcome [122,123,124]. This led to the development of the DecisionDX–PRAME test as an add-on to the DecisionDX–UM GEP test, since a Class 1 patient positive for PRAME has an increased risk of metastasis, while a PRAME-positive Class 2 patient may experience a shorter time to metastasis [122,123,124]. UM tumors with black pigmentation exhibited a higher expression of PRAME, and 70% of metastases demonstrated PRAME expression [125]. Additionally, a new oncogenic role for PRAME has been unveiled, wherein it activates meiotic genes, leading to chromosomal and genomic instability [126]. PRAME expression also leads to the ubiquitination of SMC1A (structural maintenance of chromosomes 1A), a key component of the cohesin complex that is crucial for aligning sister chromatids during homologous recombination, as well as in maintaining telomeres and ensuring proper chromosome segregation [126,127]. This ubiquitination process disrupts SMC1A interaction with STAG2 (stromal antigen 2), another vital element of the cohesin complex [128]. This disruption has significant implications, as it compromises the integrity of critical cellular processes, potentially contributing to the development and progression of cancer. The newly described functions of PRAME open new avenues for investigating its role in UM progression and for leveraging therapeutic vulnerabilities created by its expression [126].

The tumor microenvironment (TME) plays a pivotal role in the development, metastatic progression, and recurrence of UM. Pro-angiogenic tumor-associated macrophages (TAMs) within the TME are instrumental in facilitating the homing, extravasation and subsequent metastasis of UM to the liver (reviewed in [129]). Indeed, in UMs with monosomy 3, TAMs are primarily of the proangiogenic M2 polarization type [130,131]. RNA sequencing data from 63 UM cases has established a M2-macrophage-specific prognostic signature: CCL18, SIGLEC7, CD300LF, CAPG, LILRA4, SDS, and FAHD2CP, associated with high-risk UM groups [132]. The expression levels of these mRNA transcripts were linked with clinical data of tumor patients, including tumor mutational load, immune checkpoints, and drug sensitivity [132]. In addition, a MUM prognostic model using immune and stromal index was constructed (*n* = 63 participants), that included a down-expression of HLA-J, MMP12, HES6, and ADAMDEC1 mRNA transcripts [133]. Recent studies have highlighted the importance of miRNAs and mRNA transcripts in predicting UM prognosis, including distinct profiles associated with better or worse survival outcomes, pointing to their potential as prognostic and therapeutic markers (reviewed in [134,135]). For example, nine differentially expressed miRNAs found in MUM exosomes, i.e., downregulated hsa-miR-191-5p and -223-3p and upregulated hsa-miR-203a, 139-3p, -122-5p, -486-5p, -144-5p, -10b-5p and -483-5p, were identified as potential progression indicators [121]. Three miRNAs, particularly the upregulation of hsa-miR-199a-3p and the downregulation of hsa-miR-1296-3p and -508-3p, were also found in high-risk UM patients [136].

Recent advancements have not only enhanced prognostic modeling but have also paved the way for more individualized treatment approaches, showcasing the utility of new immune gene signature biomarkers, such as IL32 (interleukin 32), IRF1 (interferon regulatory factor 1), SNX20 (sorting nexin 20) and VAV1 (vav guanine nucleotide exchange factor 1), related to UM survival and disease progression [137,138]. Alongside these immunological insights, novel computational techniques have emerged. Integrated multi-layered molecular networks (iUMRG) enable the identification of high-confidence susceptibility genes (HSGs) and potential drugs, opening doors to tailored strategies for diagnosis, prognosis and treatment in specific cancers, including UM [139]. The identification of specific gene and miRNA signatures associated with metastasis and survival emphasizes the move towards personalized therapy. CXCR4 (C-X-C chemokine receptor 4) is known to be critical in the spread and extraversion of diverse cancer cell types, significantly contributing to the development of liver metastases (reviewed in [140]). In UM, it has been shown that elevated levels of CXCR4 serve as a molecular biomarker for liver metastases (reviewed in [141]). Therefore, a non-invasive staging method, such as the CXCR4-targeted magnetic resonance imaging (MRI) contrast agent ProCA32.CXCR4, has been developed to facilitate the early detection of small, stage 2 liver metastases transitioning from dormancy to activation in various metastatic murine models [142]. With additional research, this novel agent is anticipated to be useful in monitoring high-risk patients, personalizing treatment strategies and assessing the effectiveness of treatments [142]. Furthermore, technological innovations, such as single-cell applications, including scRNA-sequencing and scDNA-sequencing, have further enriched the field, allowing researchers to probe UM invasiveness and heterogeneity [143]. Indeed, one scRNA-seq study has investigated the TME and CNV, from a mix of eight primary UM and three metastatic UM samples. They have shown that tumor cells primarily clustered according to a GEP-based clinical prognostic classifier, and can be divided into two groups: class 1 (*BAP1* wild-type) and class 2 (*BAP1* mutant) tumors [144]. Variations in cellular composition were observed and provided evidence for ongoing genomic evolution within tumors with an increasing complexity from class 1 to class 2 tumors [144]. These findings provide deep insights into the cellular and molecular complexity of the TME, highlighting the importance of single-cell resolution analyses in understanding tumor biology and the prognostic implications of genetic markers.

In summary, this section underscores the major progress in UM research, showcasing a spectrum of novel biomarkers from miRNAs to gene signatures. These developments enrich our comprehension of UM molecular dynamics and open avenues for refined prognostic tools and targeted treatments. However, it is crucial to note that, while these newly proposed biomarkers for UM diagnosis, prognosis and personalized therapies are promising, they are still in the developmental phase and necessitate further research for their validation and practical application in clinical settings.

## 4. Altered Molecular Mechanisms in UM

Dysregulated signaling pathways play a significant role in the initiation and progression of cancer (reviewed in [145,146,147,148]). In the complex landscape of UM molecular mechanisms, various signaling pathways, including the JAK/STAT, mTOR, β-catenin, and autophagy, as well as transcription factors, play a pivotal role in disease progression and patient survival. These pathways influence cell growth, survival, differentiation, and tumorigenic properties. Findings related to hypoxia, methylation, and inflammatory signaling highlight the multifaceted interplay between gene regulation, metabolic pathways, immune functions, and TME in UM. Overall, this section accentuates the intricate UM molecular mechanisms and the critical importance of specific pathways in understanding the disease complexity and potential targeted therapies (Table 1).

### 4.1. Metabolic Pathways

Cancer progression and survival depend on altered metabolic pathways, allowing tumor cells to adapt, proliferate, and resist therapeutic interventions (reviewed in [149]). The strategy of focusing on cancer cell metabolism for treatment is showing great promise, and there is increasing evidence that identifying metabolic heterogeneity is crucial for determining the effectiveness of cancer therapies [150,151]. UMs with *BAP1* mutations showed an increase in oxidative phosphorylation (OXPHOS) gene set expression, such as GLUT3 (glucose transporter 3), HK1 (hexokinase 1) and CPT1A (carnitine palmitoyltransferase1A) [152]. Metabolic heterogeneity was identified within these *BAP1* mutant samples, revealing two distinct metabolic phenotypes: OXPHOS^high^, characterized by elevated glycolysis and nucleotide biosynthesis (GLUT3 and HK1), and OXPHOS^low^, which relies heavily on fatty acid oxidation (CPT1A) [152]. Additionally, protein expression patterns in the vitreous humor revealed a notable prevalence of metabolic processes, including glycolysis, gluconeogenesis and amino acid biosynthesis, particularly in GEP class 2 UM cases [115]. Along with the metabolic pathways, alterations in cellular signaling were also discovered. Bulk RNA-sequencing analysis revealed a change in SPP1-CD44 signaling, with SPP1 (also known as osteopontin) being a member of the small integrin-binding ligand N-linked glycoprotein family (reviewed in [153]), and CD44, functioning as a non-kinase transmembrane glycoprotein (reviewed in [154]) [155]. An OS prognostic signature was defined using five metabolism-related genes (MRGs): MDH2 (malate dehydrogenase 2), NME1 (nucleoside diphosphate kinase 1), NT5C2 (5‘-nucleotidase, cytosolic II), which are linked to high-risk UMs, while PC (pyruvate carboxylase) and ENPP1 (ectonucleotide pyrophosphatase/phosphodiesterase 1) were associated with low-risk UMs [156]. Further relationships were found between S100A13 expression and the ROS pathway [157], and an elevated expression of HO-1 (heme oxygenase 1) was observed in UM cell lines, suggesting a correlation with enhanced cell proliferation and UM progression [158]. The significant increased expression of PNPO (pyridoxine 5′-phosphate oxidase), an essential enzyme in vitamin B6 metabolism, has been observed in twenty-one types of tumors, including UM, suggesting a potential role in facilitating immune evasion during immunotherapy [159]. Furthermore, the AMPK (adenosine monophosphate-activated protein kinase) signaling pathway, a crucial cellular energy-sensing and regulatory pathway (e.g., mTOR signaling), was found downregulated in UM cells compared to normal choroidal melanocytes, with this regulation being dependent on *BAP1* and mediated through CaMKK2 (calcium/calmodulin-dependent protein kinase kinase 2) and potentially LKB1 (liver kinase B1) [160,161]. Moreover, SIRT5 (sirtuin 5), a key player in multiple metabolic pathways, influences the proliferation and survival of both UM and CM [162]. The depletion of SIRT5 led to a significant loss of cell proliferation and increased cell death in both UM and CM cell lines in humans and mice across various genetic backgrounds [162]. Lastly, a metabolic-related gene signature could predict the prognosis of UM patients with the increased expression of SYNJ2 (synaptojanin 2) and CA12 (carbonic anhydrase XII) linked to a higher risk, while the elevated expression of ABCA12 (ATP binding cassette subfamily A member 12) and SLC25A38 (solute carrier family 25 member 38) correlated with a lower risk [163]. This signature reflects a dysregulated metabolic microenvironment and suggests new metabolic biomarkers and therapeutic targets for UM [163].

### 4.2. Autophagy and Mitophagy

In the ongoing exploration of genetic factors influencing cancer, several studies have unveiled the critical role of gene signatures linked to autophagy and mitophagy (reviewed in [164,165]) [166]. Autophagy, a process crucial for maintaining energy and recycling nutrients, plays a dual role in cancer development. While it prevents cancer in normal cells by removing damaged components and reducing harmful substances, in tumor cells, it paradoxically enhances metabolism, nutrient uptake, and drug resistance, thereby promoting cancer progression (reviewed in [167]). Previous studies have demonstrated that, in UM patients, there is a frequent overexpression of autophagy-related proteins, such as MAP1LC3A (microtubule-associated protein 1 light chain 3 alpha) and BECN1 (beclin 1), which are associated with tumor progression and poorer outcomes [168]. Increased autophagy in UM cell lines contributes to tumor cell survival under stress, highlighting the significant role of autophagy in UM progression [169]. Recently, the dysregulation of nine autophagy-related genes (low expression of TUSC1, LMCD1, GABARAPL1, PRKCD, DLC1, and high expression of FKB1A, ITGA6, BNIP1, IKBKE) was examined [170]. These mRNA transcripts were found to correlate with high-risk molecular characteristics and had a substantial impact on OS rates [170]. Adding further complexity to the understanding of UM, the expression levels of several autophagy genes in patients who experienced metastasis were analyzed and uncovered a six-gene signature comprised of long non-coding RNAs (lncRNAs), which not only correlated with OS but also provided effective prognosis predictions for UM patients [171]. Additional evidence points to six specific autophagy-linked lncRNAs that exhibited differential expression in UM cell lines with lncRNAs, SOS1-IT1, AC016747.1, AC100791.3 and AC018904.1 acting as risk factors, whereas AC104825.1 and AC090617.5 serving as protective elements [172]. Lower expression of LINC01278 was linked to high-risk UMs and suppressed the proliferation, migration and invasion of UM cells by promoting autophagy [173].

Mitophagy, a unique form of autophagy specific to mitochondria, selectively eliminates damaged and aged mitochondria. This process is crucial for maintaining both the quantity and quality of mitochondria within cells (reviewed in [174]). Interestingly, mitophagy in cancer cells has a double function: it prevents tumorigenesis by removing dysfunctional mitochondria in early stages but, in established tumors, it aids in cancer cell survival and proliferation by reducing oxidative stress and recycling substrates (reviewed in [175]). Recently, a four-gene signature (PGAM5, SQSTM1, ATG9A, GABARAPL1) related to mitophagy was identified, revealing its predictive patients’ survival value across various cancer types, UM included [176].

### 4.3. mTOR and β-Catenin Signaling Pathways

The mTOR and β-catenin signaling pathways are central to the regulation of cell growth, survival, and differentiation in various types of cancer, as well as UM (reviewed in [11,12,13]). The mTOR signaling pathway primarily involves downstream effectors, like AKT (protein kinase B), S6K (S6 kinase) and 4E-BP1 (4E-binding protein 1), which regulate UM crucial cellular processes, such as cell growth and protein synthesis [177]. The β-catenin pathway activates transcription factors such as TCF/LEF, influences gene expression through targets like c-MYC and cyclin D1, and regulates its own degradation via AXIN2 (axis inhibition protein 2) and GSK3β (glycogen synthase kinase 3 beta) [178]. In recent years, circular RNA (circRNA) circ_0119872, a type of noncoding RNAs characterized by its continuous loop structure lacking open 3′ and 5′ ends [179], was identified as a UM oncogene by sequestering miR-622, leading to the suppression of G3BP1 (GTPase-activating protein SH3 domain-binding protein 1) expression and, the stimulation of the Wnt/β-catenin and mTOR signaling pathways [180]. Parallelly, ZNF704 (zinc finger protein 704) overexpression promotes the growth and migration of UM cells, with its downregulation leading to dysregulation of SORBS3 (sorbin and SH3 domain containing 3) and activation of the PI3K/AKT/mTOR pathway, epithelial-mesenchymal transition (EMT), and metastasis-related genes [181]. The FASN (fatty acid synthase) expression, controlled by the mTOR–SREBP1 (sterol regulatory element-binding protein 1) axis, is upregulated in a choroidal melanocyte line transduced with *GNAQ^Q209L^* and in UM cells mutated in *GNAQ*/*GNA11* [182]. This study also shows that inhibiting FASN and mTOR not only suppresses UM cell growth but also induces cell cycle arrest and apoptosis [182]. Furthermore, ZNF704 operates through the AKT/mTOR/glycolysis signaling pathways, and the restoration of UM tumor cell viability in ZNF704-silenced cells can be achieved by knocking down SORBS3 [181]. In a broader context, it is important to note that high expression of mTORC1 signaling, related to these pathways, has been associated with low OS in UM patients, underlining the clinical significance of these molecular interactions [183]. Along with the reduced AMPK signaling, mTOR activity was also observed to be lower in UM cells compared to normal choroidal melanocytes, influenced by the energy-dependent LKB1 (liver kinase B1)-AMPK pathway [160]. The kinase LKB1, essential for UM cell proliferation, has been demonstrated to be necessary, with its expression being regulated by HGF (hepatocyte growth factor) [184].

### 4.4. Inflammatory Signaling Pathways

Within UM, several inflammatory signaling pathways, including IL6-JAK-STAT3, IL2-STAT5, INF-α/γ, and TNF-α pathways, exhibit significantly higher hazard ratios [185]. Inflammation-related molecules, such as NF-κB (NFKB1), COX-2 (PTGS2) and CXCL10 (C-X-C motif chemokine ligand 10), primarily expressed in the macrophages, were linked to a poor prognosis in UM (reviewed in [186]) [185]. *BAP1*-mutant UMs were found to suppress the NF-κB signaling pathway, therefore creating an immunosuppressive microenvironment by decreasing cytokine secretion and antigen-presenting capacity by macrophages [187]. A distinctive signature consisting of nine inflammatory response-related mRNA transcripts (PDE4B, RAF1, CXCL8, P2RX4, LPAR1, ITGA5, CCL24, ITGB3, CCL20) has been correlated with UM survival, highlighting ITGA5 (integrin alpha 5) and P2RX4 (P2X purinoceptor 4) as key mRNA transcripts [188]. Within the high-risk prognosis group of UM with metastasis-associated genes, there is a notable increase in the activation of antigen-presenting cells (APC) stimulation, checkpoint signaling, HLA (human leukocyte antigen) and type II-IFN (type II interferon) response [189]. Additionally, four out of the top nine methylation-regulated mRNA transcripts (EDNRB, IL12Rβ2, CALHM2, RNF43) have been associated with immune functions, antitumor activity and UM survival [190]. TME remodeling has been linked to pyroptosis, an inflammation created by programmed cell death, where upregulation and hypomethylation of pyroptosis-related genes predicted poor survival in UM [191].

### 4.5. Gene Expression Pathways

The functional impairment of *BAP1* has been linked to widespread chromatin compaction. This polycomb-mediated gene repression is characterized by the spreading of the histone modification H2AK119ub1, where one ubiquitin molecule is attached to the 119th lysine residue of histone H2A, and an elevation in H3K27me3, where three methyl groups are added to the lysine residue at position 27 of histone H3 [192]. Deregulation of N6-methyladenosine (m^6^A) RNA methylation, which is the methylation at the adenosine base’s sixth position and the most common internal change in mRNA mainly found in 3‘ untranslated regions (3‘UTRs) [193], has been found to promote and contribute to UM genesis (reviewed in [194]). Lastly, aberrant DNA methylation drives transcriptomic changes and is linked to a poor cancer prognosis [77,195]. This abnormal DNA methylation, relatively uniform across the entire genome, disrupts essential oncogenic pathways, including those related to EGFR tyrosine kinase inhibitor resistance, focal adhesion, proteoglycans in cancer, PI3K-AKT signaling and ECM-receptor interactions [77]. Quantitative mass spectrometry analysis of UM tumors has uncovered specific histone post-translational modifications (PTMs) associated with *BAP1* status, as well as tumor stage and grade, such as increased levels of H3K4me1, peptides with H3K9me3, histone H3 27-40 with K36me2 and K27 methylations, multi-acetylated H4 tails, and H4K20me3 [196].

### 4.6. Hypoxia

Hypoxia, a diminished oxygen level, is a characteristic element in the growth of malignant tumors, including UM (reviewed in [197]). The adaptation to hypoxia is orchestrated by various transcription factors, chiefly HIF-1 (hypoxia-inducible factor 1), which acts as the primary oxygen sensor and central regulator of gene responses triggered by low oxygen conditions (reviewed in [198]). UM patient biopsies and Gene Set Enrichment Analysis (GSEA) revealed a unique molecular signature, which includes HIF-1α and other factors, as a precise indicator for predicting UM metastasis [199]. The presence of hypoxic UM tumors has been correlated with an increased risk of metastasis, aggressive phenotypes, and poor clinical outcomes, including more *BAP1* mutations and loss of one copy of chromosome 3 [200]. These tumors have shown a connection with the highest hazard ratios and the lowest OS rates [185]. Along with the association of hypoxia-related genes with aerobic respiration [200], there is also an observed elevation in the levels of hypoxia-regulated mRNA transcripts P4HA1 and P4HA2, whose protein encoded products are implicated in proline-hydroxylated collagen secretion and deposition in the extracellular matrix, contributing to negative prognosis in MUM [201]. This upregulation can be counteracted by the compound KCN1, a hypoxia-inducible inhibitor, illustrating a potential therapeutic approach targeting hypoxia influence on UM progression [201].

### 4.7. MAPK Pathway

Activating mutations in *GNAQ*/*11* genes are prevalent in approximately 85% of UM cases, leading to the activation of the MAPK pathway. This aberrant signaling drives UM progression and contributes to its malignancy [42]. There is growing evidence indicating that STING (stimulator of interferon genes, also known as TMEM173) plays a role in the development and spread of many tumors including UM [202,203,204]. STING encodes for a transmembrane protein found in the endoplasmic reticulum and mitochondria, which is present in tissues associated with the immune system, as well as some malignancies and tumors (reviewed in [205]). Not only is it more abundantly expressed in UM tissues compared to adjacent healthy tissues, but STING also enhances the invasion and migration of UM cells by increasing the activity of the p38-MAPK signaling pathway [204].

### 4.8. JAK/STAT Pathway

The JAK/STAT signaling pathway, a critical mediator in various cellular functions, has emerged as a significant player in the progression of cancer, including UM, where its aberrant activation has been linked to increased growth, survival, and malignancy of tumor cells [9,10]. In UM GEPs, the HTR2B (5-hydroxytryptamine receptor 2B) stands out as the most dysregulated mRNA transcript in high-risk MUMs and has connections with the JAK/STAT pathway. Studies have demonstrated that STAT proteins augmented HTR2B expression positively in UM cell lines [206]. In an integrative multi-omics analysis of UM samples (*n* = 80) with loss of chromosome 3 called M3 iSubtype (iCluster; indicative of the worst survival), the IL6/JAK/STAT3 signaling pathway was identified to be hypomethylated and increased in M3 tumors, in conjunction with other pathways like angiogenesis, allograft rejection, inflammatory response IFN-γ response [207]. Furthermore, the high activation of the IL6/JAK/STAT3 signaling pathway was correlated with a decrease in OS rates [183].

### 4.9. Other Molecular Mechanisms

HDAC7 (histone deacetylase 7) overexpression has been observed in UM in comparison to normal tissues, a mechanism that led to increased proliferation and metastasis mediated by c-MYC [208]. KIT overexpression has been linked to a poor prognosis in cases with monosomy 3 [209], and aberrant expression of PDCD2L (programmed cell death 2 like) has been observed across various types of cancers, including UM [210]. Another significant discovery pertains to HES6 (hairy/enhancer of split family basic helix-loop-helix transcription factor 6), identified from scRNA-sequencing data as a key driver for MUM [211]. HES6 exhibits crucial tumorigenic properties, functioning downstream of the NOTCH signaling pathway and affecting the motility of primary UM cells [211]. Furthermore, the role of EMT has been highlighted as a determining factor in outcomes for MUM patients, particularly those with an OS of less than a year [183]. In this context, NRP1 (neuropilin-1) is associated with EMT in multiple tumor types [212,213,214,215]. This glycoprotein was shown to bind to various vascular endothelial growth factor isoforms, as well as TGF-β1, and has been directly correlated with survival rates of less than 1 year in UM patients [183].

**Table 1:** Overview of altered UM molecular mechanisms and risk factors. This table highlights key genes and findings, their impacts on UM pathogenesis and progression, and associated risk categories. Each row details a different molecular pathway or mechanism, summarizing recent discoveries and insights into how they influence UM progression. It also identifies whether they are associated with high-risk or low-risk UMs, based on current research findings.

Pathways/Mechanisms	Key Findings/Genes	Impact in UM	Risk Categories	References
Metabolic pathways	*BAP1* mutations leading to OXPHOS gene set expression variations (GLUT3, HK1, CPT1A)	Alteration of cancer cell metabolism, contributing to therapeutic resistance	High-risk: OXPHOS^high^ Low-risk: OXPHOS^low^	[152,156,157,158,159,160,161,162,163]
mTOR and β-catenin signaling pathways	CircRNA circ_0119872, FASN, ZNF704, SORBS3, LKB1 regulated by HGF	Regulation of cell growth, survival and differentiation	High-risk: associated with low survival	[177,178,179,180,181,182,183,184]
Inflammatory signaling pathways	IL6-JAK-STAT3, NF-κB (NFKB1), COX-2 (PTGS2), CXCL10, nine-gene inflammatory signature including ITGA5 and P2RX4	Influence on immune microenvironment and tumor progression	High-risk: associated with poor prognosis Low-risk: ITGA5 and P2RX4	[185,186,187,188,216]
Autophagy and mitophagy	Gene signatures related to autophagy and mitophagy; Autophagy-linked lncRNAs	Correlation with UM survival rates and molecular characteristics	High-risk: dysregulated autophagy genes	[168,169,170,171,172,191]
Gene expression pathways	*BAP1*, m^6^A RNA methylation, DNA methylation, EGFR resistance, histone PTMs	Effects on chromatin compaction, gene expression and resistance pathways	High-risk: abnormal DNA methylation and histone PTMs	[192,194,195,196]
Hypoxia	Hypoxia-regulated genes: P4HA1, P4HA2	Association with metastasis, aggressive phenotypes, and poor outcomes	High-risk: hypoxic tumors	[197,199,200,201]
MAPK pathway	Activating mutations in *GNAQ*/*11* STING high expression	Activation of MAPK pathway, driving UM progression Enhancement of UM cell invasion and migration; increase of the activity of p38-MAPK signaling	High-risk: activated MAPK pathway	[42,204]
JAK/STAT pathway	HTR2B, IL6/JAK/STAT3 and related pathways	Aberrant activation linked to increased tumor growth and malignancy	High-risk: dysregulated JAK/STAT pathway	[206,207]
Other molecular mechanisms	HDAC7, KIT, PDCD2L, HES6, NRP1, EMT factors	Various roles in proliferation, metastasis, and survival	High-risk: overexpression of HDAC7, KIT, PDCD2L, NRP1	[208,209,210,211]

## 5. Immune Microenvironment

The UM immune microenvironment presents a complex and unique landscape that is central to understanding the disease behavior and progression. Influenced by factors such as the anatomical position with the presence of the blood-retinal barrier and its capacity to modulate the immune system, the UM immune microenvironment plays a critical role in its tendency to metastasize primarily to the liver [185,217]. The following sections delve into the intricate aspects of this environment, exploring elements such as CD8^+^ tumor-infiltrating lymphocytes (TILs), immune-related gene expression, noncoding RNAs, immune subtypes, and more. These insights provide valuable information for the development of personalized treatments, prognostic, and diagnostic strategies for UM patients, emphasizing the need for a comprehensive understanding of immune interactions within this specific cancer.

### 5.1. Gene Expression and Immune Responses

In high-risk UMs, the aggressive behavior of the tumor is underpinned by several complex factors. Key among these is the enrichment of pathways tied to immune evasion and metastasis. Distinct genes governing tryptophan metabolism and the function of MMPs have been identified, showing varied expression across different risk profiles and underlining a unique biological framework [188,218]. Heme proteins TDO (tryptophan 2,3-dioxygenase) and IDO (indoleamine 2,3-dioxygenase) catalyze the conversion of tryptophan into kynurenine by relying on GAPDH (glyceraldehyde-3-phosphate dehydrogenase) expression and its heme binding ability [219]. High expression of the TDO enzyme was found in stage IV UM patients (*n* = 16) with a stronger signal in hepatic metastases than surrounding healthy hepatocytes, in contrast with IDO that was not detectable [220]. Since kynurenine can inhibit T cell function, TDO is thought to impact the UM patients’ outcome by contributing to cancer immune escape [220]. Moreover, the UM tumor immune microenvironment (TIME) is sculpted by a sophisticated interplay involving gene signatures, hypoxia, chemokines and immune-related genes [157,170,185,200,221]. TIME is a key factor in the advancement of cancer and its resistance to treatment and is linked with UM genomic alterations [222]. For instance, the loss of *BAP1* has been related to an immunosuppressive tumor microenvironment via the PROS1/MERTK ligand, which activates immunosuppressive CD163^+^ macrophages [223]. Furthermore, the interaction between UM tumor cells and CD8^+^ T-cells has been associated with poor prognostics and was stronger in *BAP1*-mutant cells using the activation of ITGB2 (integrin subunit beta 2) and ICAM1 (intercellular adhesion molecule 1) [224]. Additional aspects, such as inflammatory pathways and alterations in B7 family expression, a group of cell surface proteins that plays a critical role in the regulation of immune responses, have been correlated with diverse characteristics in UM, including cytotoxic T-cell levels and methylation patterns [185,225]. Interestingly, STEAP1 (six transmembrane epithelial antigen of the prostate 1) exhibits high expression levels in various types of cancers, including bladder, colon, ovarian and prostate, playing a significant role in enhancing the invasive capabilities of tumor cells [226]. However, in UM, it has been linked to the modulation of immune-infiltrating neutrophils and its elevated expression was associated with a favorable prognostic [227]. Dysregulation in immune pathways and HLA expression in high-risk UMs also contributes to an enhanced propensity for metastasis [171]. Patients with lower risk scores showed a higher infiltration proportion of CD8^+^ T-cells and a lower infiltration percentage of regulatory T-cells [228]. Macrophages M1 and M2 were two subtypes that play inverse functions. Indeed, in low-risk patients, an elevated infiltration of M1 macrophages and a decrease in immunosuppressive M2 macrophages were observed [228]. Moreover, hypomethylation and increased expression of CD3D (CD3 delta subunit of T-cell receptor complex) promoted the infiltration of immune cells, as well as the proliferation, migration and invasion of UM cells, thereby accelerating the progression of UM [195]. Recently, attention has been drawn to the potential of natural killer (NK) cells to exhibit immune regulatory properties, in addition to their well-known capacity for antitumor activity [229,230]. A recent study revealed that a higher frequency of NK cells and an elevated expression of the TNF superfamily member 4-1BB ligand are linked to a worse prognosis, indicating their ability to adopt a pro-metastatic role in UM [231]. These multifaceted interactions influence the tumor behavior and response to therapy, providing crucial insights into disease progression and potential therapeutic targets.

### 5.2. TILs in High-Risk UMs

TILs consist of a diverse array of immune cells, primarily comprising T, B, NK, dendritic and myeloid cells (reviewed in [232]). Various studies suggest that, in UM, increased CD8^+^ T-cells and reduced PD-L1 (programmed death-ligand 1) expression are associated with a poorer prognosis [232,233,234,235,236]. In a recent study, a prognostic risk model was developed to pinpoint co-expressed mRNA transcripts that facilitate the infiltration of CD8^+^ T-cells, using four mRNA transcripts: PTPN12 (protein tyrosine phosphatase non-receptor type 12), IDH2 (isocitrate dehydrogenase 2), P2RX4 (purinergic receptor P2 × 4) and KDELR2 (KDEL (lys-aspp-glu-leu) endoplasmic reticulum protein retention receptor 2) [237]. These four co-expressed mRNA transcripts primarily contribute to the infiltration of CD8^+^ T-cells by improving antigen processing and presentation, and their expression was associated with a poor prognosis [237]. Furthermore, the connection between the expression of HNRNPCs (heterogeneous nuclear ribonucleoproteins C), known for their role in regulating alternative splicing as RNA-binding proteins, and CD8^+^ T-cell infiltration showed an almost perfect correlation with UM [238]. This counterintuitive finding adds to the complexity of understanding UM immune landscape and suggests the need for further investigation into the role and behavior of TILs.

### 5.3. Immune Subtypes (IS) and Their Prognostic Significance

The TCGA–UM gene expression data have played an important role in unraveling the complex relationship between tumor cells and the immune system. This data set (*n* = 80) was used to formulate a new classification system centered on immune-related genes and established an immune-based prognostic indicator, resulting in the identification of immune subtype (IS) clusters: IS1, IS2 (A and B) and IS3, which were notably associated with differences in OS and progression-free survival (PFS) respectively from the worst to the best [137]. Particularly, IS3 stood out for its favorable prognostic implications and sensitivity to a PD-1 inhibitor, while IS1 was the most immunosuppressive subtype [137,239]. High-risk UMs have been associated with infiltration of specific immunocytes and high expression of particular genes linked to prognosis [189]. High-risk UM patients exhibit a higher expression of immune checkpoint genes and an enrichment of immune-related markers, indicating potential targets for immunotherapy [240]. Recent research offers insights into the complex interplay between genetics, immune response, and molecular pathways in UM. Indeed, the presence of clonally expanded T-cells and plasma cells in UM samples indicates an active immune response, challenging the belief that the poor response to checkpoint inhibitors is solely due to low tumor mutation burden [144]. The failure of therapies targeting CTLA-4 (cytotoxic T-lymphocyte associated protein 4) and PD-1 (programmed cell death protein 1) in UM could be explained by LAG3 (lymphocyte-activation gene 3) being the dominant immune exhaustion marker [144]. Understanding these multifaceted interactions is essential for patient prognosis and the development of targeted treatment strategies. This comprehensive view brings to light the unique immune microenvironment in UM, with implications for diagnosis, prognosis, and therapeutic interventions.

## 6. Current and Emerging Therapeutics

The main treatment methods for primary UM encompass radiotherapy (either plaque brachytherapy or external radiation therapies), transpupillary thermotherapy, and various forms of tumor removal, including transscleral resection, endo-resection and enucleation (reviewed in [3,241]). Regrettably, around 50% of individuals initially diagnosed with UM eventually experience progression to MUM, predominantly affecting the liver (about 89%) (reviewed in [3,5,7]). This progression is linked with a grim survival outlook, with median OS times varying between 4 and 15 months (reviewed in [3,5,7]). There is no established standard treatment for MUM, and the available therapeutic choices offer limited advantages. MUM patients can receive additional treatments such as localized resection, immunotherapy (Ipilimumab and Pembrolizumab), chemotherapy (Dacarbazine) and therapy targeting specific molecular markers (reviewed in [4]). Unfortunately, these approaches are very ineffective due to the fact that it is frequently transposed from CM research (reviewed in [242,243]). However, the FDA approval of KIMMTRAK in 2021 (Tebentafusp, IMCgp100), a novel bispecific immunotherapeutic agent targeting gp100 and the CD3 protein complex on T-cells, marks it as the first drug to substantially extend survival in MUM patients (commented in [244,245] and reviewed in [246]). Nonetheless, it remains crucial to discover new treatments for MUM patients that are both highly effective and long-lasting, since only a specific group of MUM patients (i.e., HLA-A*02:01-positive) are eligible to this immunotherapy; for these patients, the 1-year OS rate improved to 73%, while the median survival increased to 21.7 months [247,248]. Therefore, the overall prognosis for this cancer type remains relatively poor and there is still a need to explore additional therapeutic strategies to further extend patient survival (Table 2).

### 6.1. Histone Deacetylase Inhibitors (HDACi)

HDACs, a group of enzymes involved in the epigenetic regulation of gene expression, function by stripping acetyl groups from lysine residues on histones and various protein targets. This activity leads to the local condensation of the chromatin structure, which, in turn, suppresses gene expression, including that of tumor suppressor genes (reviewed in [249]). Given that HDACs are found to be abnormally expressed in UM [250,251], there is potential to targeting these epigenetic regulators in a treatment approach [252] (reviewed in [253]). A phase II trial known as PEMDAC (ClinicalTrials.gov: #NCT02697630) involving twenty-nine UM patients evaluated a treatment combination of an immune checkpoint inhibitor (Pembrolizumab) and an HDACi (Entinostat). In this trial, twelve out of twenty-nine MUM patients exhibited either a partial response or stabilization of the disease [254,255]. Quisinostat, another HDACi, modulates the immune response in UM cell lines by improving the ability of tumor cells to present antigens by increasing expression of MHC-I and its presence on the cell surface [256]. Furthermore, the use of the HDACi Ricolinostat (ACY-1215) on a MUM cell line led to the interruption of cell cycle progression in S phase and triggered apoptosis [257]. Additionally, the newly synthesized HDACi VS13, which targets HDAC6 with a nanomolar affinity, demonstrated potent antiproliferative effects and effectively halted the cell cycle in the G0/G1 phase in UM cells [258].

### 6.2. MAPK Signaling Pathway Inhibitors

Therapeutic agents targeting the Gαq canonical signaling pathway PLCβ–PKC–MAPK have shown minimal impacts on the OS of patients with MUM, whether used as single agents or in combination with chemotherapy [259,260]. A phase I/II clinical trial (#NCT03947385) involving the PKCi darovasertib (IDE196), both as a standalone treatment or in combination with Crizotinib, a multi-kinase inhibitor, or Binimetinib (MEKi), is still ongoing. Using this treatment, 9.1% of patients (*n* = 6 of 66 participants) responded positively, with complete or partial responses [261]. Three other clinical trials are currently recruiting for IDE196 alone (#NCT05907954 (phase II), #NCT05987332 (phases II/III), and #NCT03947385 (phases I/II)). Moreover, initiation of enrollment for phase II of the clinical trial #NCT01801358, using PKCi and MEKi, did not proceed due to both limited clinical efficacy and serious adverse events [262]. The novel PKCi LXS196 was tested in a phase I clinical trial (#NCT02601378) and showed manageable toxicity compared to IDE196 and encouraging clinical activity as a single agent, where 67% of UM patients had stable disease progression [261]. This led to the development of NVP-LXS196, an optimized LXS196 broadly-targeting PKC with a high selectivity across the entire kinome [263]. A novel focus has emerged by directly targeting Gαq. Indeed, a study showed that UM cells with activating mutations in one of three residues in *GNAQ*, as well as wild-type Gαq driven by *CYSLTR2^L129Q^*, were highly sensitive to a combination treatment using a Gαq inhibitor with a MEKi [43]. Additionally, a new small molecule, identified as F33, has been formulated featuring a quinazoline structure. This compound demonstrated strong inhibitory effects on Gαq/11 proteins and exhibited anti-proliferative activity against two UM cell lines, MP41 and 92.1 [264]. Interestingly, a study investigated the feasibility of administering a genetically encoded inhibitor of Gαq (1EBB25) to HEK293 cells, and showed a strong inhibition for transient expression of Q209P and Q209L Gαq variants [265]. Current work is testing the inhibitor’s efficacy in UM cells that exhibit Gαq mutations, aiming to hinder tumor proliferation [265]. Lastly, the revelation of a tumor-suppressing miRNA that specifically targets GNAQ and AKT3 (miR-181a-5p) proved effective in hindering the progression of UM [266]. Even though targeting these proteins showed great promise, additional research is required to establish its clinical relevance.

### 6.3. Hippo/YAP Pathway Inhibitors

There is still debate in the field as to which YAP pathway is the most important for the tumorigenic potential of *GNAQ*/*11*. YAP is not mutated in UM but many tumors display the activated, nuclear form [267,268]. However, another study reported no correlation between nuclear expression of YAP and patient survival [269]. A recent study in the zebrafish model showed that hyperactive ERK may be dispensable for UM pathogenesis, and demonstrated that YAP was sufficient to lead to tumor growth and was active when *GNAQ*, *CYSLTR2* and *PLCβ4* were mutated, thus suggesting that targeting YAP could be a more promising therapeutic strategy for UM than PKC/MEK/ERK inhibitors [53]. Activating mutations in *GNAQ*/*11* has been shown also to trigger a non-traditional pathway that activates the Hippo/YAP pathway, which involves TRIO-RhoA and FAK (focal adhesion kinase), presenting more promising therapeutic opportunities [270,271]. It was shown that a combination of FAKi (VS-4718) and MEKi (Trametinib) was synergistic in both in vitro and in vivo UM models [271]. Furthermore, treatments combining FAKi with either MEKi or PKCi exhibited a strong synergy, markedly lowering cell viability and promoting apoptosis [272]. This combination also showed remarkable in vivo efficacy in UM patient-derived xenografts (PDXs) [272]. Lately, the strong synergy observed from simultaneously using FAKi (VS-4718) and PKCi (Darovasertib), in in vitro or xenografted MUM models, led to cell death and the regression of tumors showing a synergistic antiproliferative effect on UM cells [273]. Considering these observations, a phase I clinical trial was started in 2020 (#NCT04109456), employing FAKi (IN10018 or Defactinib), first used alone, then in conjunction with MEKi (Cobimetinib or VS-6766) and a PD-L1 checkpoint inhibitor (Atezolizumab) for 120 MUM patients. Additionally, a phase II clinical trial was started in 2021 (#NCT04720417), evaluating the combination of FAKi Defactinib (VS-6063) and MEKi VS-6766 (CH5126766) in thirteen MUM patients. For these clinical trials, no results have been disclosed and their primary completion date is due mid-2024.

### 6.4. Advanced Drug Delivery Systems

A newly designed nanoparticle (NP^PDT^) was created to deliver 56MESS ([5,6-dimethyl-1,10-phenanthroline] [1S,2S-diaminocyclohexane] platinum [II]), a chemotherapeutic agent that generates reactive oxygen species (ROS) when excited at 808 nm [274]. The combined effects of ROS and 56MESS efficiently reduced UM cell proliferation and in vivo tumor growth by damaging cellular DNA and mitochondria [274]. Additionally, it stimulated the cGAS-STING pathway, a natural immune signaling pathway that activates and increases immune infiltration in the TME, thereby triggering specific antitumor immune responses [274]. In addition, a phase II compound Belzupacap sarotalocan (AU-011), a virus-like drug conjugated with a photosensitizer (phthalocyanine), was tested and tailored for a first-line UM treatment (#NCT03052127 and #NCT04417530) [275]. It is distinctive due to its tumor-targeting capabilities via HSPGs (heparan sulfate proteoglycans), that are overexpressed on tumor cells [275]. Preliminary AU-011 research showed promise in triggering immunogenic cell death in various UM cell lines [276]. This treatment appears to work as an immuno-stimulant by increasing the exposure of DAMPs (damage-associated molecular patterns) on the cell membrane, specifically CRT (calreticulin) and HSP90 (heat shock protein 90) [276].

For MUM patients with unresectable liver metastases, the use of selective internal radiation therapy (SIRT), also known as radioembolization, can be applied and is a form of internal radiation therapy used primarily to treat liver tumors by delivering radiation directly to the tumor site [277]. A phase II clinical trial (#NCT01473004) that enrolled MUM patients (*n* = 48) is testing ^90^Y-microspheres (SIR-Sphere^®^) as a treatment for liver metastases. Using radioactive Yttrium-90-(^90^Y)-microspheres showed promise when used as a first-line therapy by increasing MUM patients OS on average by a duration of 6 months [278,279]. Recently, an innovative dosimetry method utilizing ^99m^Tc-macroaggregated albumin (^99m^Tc-MAA) with single photon emission computed tomography (SPECT)/computed tomography (CT) imaging was successful in accurately forecasting the actual radiation dose delivered to tumors prior to undergoing SIRT [280]. These findings could have significant implications for improving the planning and effectiveness of SIRT treatments. Finally, a study showed that the combined use of at least 5 Gy irradiation radiotherapy with electrochemotherapy (1–2.5 µg/mL bleomycin and 750–1000 V) in UM tridimensional cell cultures significantly improved the penetration and effectiveness of the radio-sensitizing agent bleomycin, and reduced the tumor cell survival to less than 10% [281]. Further research is required to evaluate the clinical significance of this combined approach.

### 6.5. Emerging Approaches

#### 6.5.1. *BAP1*-defective Cancers

In UM, *BAP1* mutations have a strong correlation with increased metastatic risk and lower survival rates, as evidenced by the presence of *BAP1* inactivating mutations in up to 84% of MUM cases [73]. An epigenetic compound library screen, a method used to identify chemical compounds that can affect epigenetic modifications within cells, was performed using isogenic *BAP1* knockout cells and found that bromodomain and extra-terminal (BET) domain family proteins inhibitor OTX015 displayed moderate to high specificity in targeting *BAP1*-deficient cells with cytotoxic effects [282].

#### 6.5.2. CAR T-Cell Therapy (Reviewed in [283])

CAR T-cells have a specially designed chimeric antigen receptor (CAR) that targets a specific protein found on the surface of tumor cells, independent of antigen presentation. CAR T-cells derived from tumor-infiltrating lymphocytes and targeting the HER2 (human epidermal growth factor receptor 2) antigen effectively eliminated UM in PDX mouse models [284]. This showed a favorable tolerance profile and exhibited antitumor activity in mice and companion dogs diagnosed with CMs and UMs [285]. These outcomes suggest that this therapeutic strategy holds promise for the treatment of melanoma that is resistant to checkpoint immunotherapy. An ongoing clinical trial in the Netherlands is administering TCR modified T-cells as a treatment for patients having melanoma (including UM) or head and neck cancers that express MAGE-C2 (melanoma-associated antigen C2) (clinicaltrialsregister.eu; EudraCT#: 2019-000657-31). No results are available yet. Recently, TYRP1 (tyrosinase related protein 1)-directed CAR-T cell therapy showed promising antitumor activity in vitro and in vivo in patient-derived UM models [286]. Preparations for a phase 1 clinical trial are underway to further investigate the efficacity and safety of this therapy [286].

#### 6.5.3. Human Endogenous Retroviruses (HERVs)

HERVs, which constitute about 8% of the human genome, are genetic remnants of ancient retroviral infections and are being investigated to determine if they can become new targets for neoantigens, particularly in patients with cancers of low mutational burden who may exhibit T-cell recognition of HERVs [287]. In MUM, HERV differential expression was observed in between UM classes, and could differentiate between tumors that will metastasize and those that will not, prior to visible metastatic spread [288].

**Table 2:** Advantages and disadvantages of current and emerging therapeutic strategies for UM treatment. Each row corresponds to a specific therapeutic approach, detailing its potential benefits and limitations.

Therapeutic Strategies	Advantages	Disadvantages	References
Histone deacetylase inhibitors	Target epigenetic regulation Enhance immune response Synergistic potential with other treatments Specific HDAC targeting	Variable efficacy Side effects and toxicity Potential for resistance development Limited long-term data Cost and accessibility	[251,253,254,255,257]
MAPK signaling pathway inhibitors	Targeted action against a crucial signaling pathway Clinical trials ongoing Tested in combination therapies	Limited efficacy shown so far Potential adverse effects Ongoing research needed for conclusive results	[259,260,261]
Hippo/YAP pathway inhibitors	Novel target for treatment Demonstrates synergistic effects with other therapies Promising preclinical results	Early-stage research Complex pathway interactions may complicate treatment Specificity and toxicity concerns	[270,271]
Advanced drug delivery systems	Enhanced targeting of tumor cells Innovative delivery techniques like nanoparticles and radioembolization Potential combination with immunotherapy	High complexity and cost Significant regulatory hurdles Limited data on long-term efficacy and safety	[274,277]
Emerging approaches	BAP1-defective cancers: Targeting with BET inhibitors shows high specificity CAR T-cell therapy: Effective in preclinical models, promising for checkpoint-resistant melanomas Human endogenous retroviruses (HERVs): Potential new targets for low-mutational cancers	BAP1-defective cancers: High metastatic risk and lower survival rates CAR T-cell therapy: Early-stage clinical trials, results pending; HLA downregulation or loss on cancer cells can limit the therapy effectiveness Human endogenous retroviruses (HERVs): Need more research to confirm clinical relevance	[282,283,284,285,286,287,288]

## 7. Challenges and Future Directions

Challenges and future directions in UM research and management have been identified, highlighting the need for improved therapies and diagnostic/prognostic approaches. Some of the key challenges and future directions include:

### 7.1. Development of Models for Preclinical Studies and Personalized Therapies

Preclinical models, such as in vitro tridimensional models (e.g., tumor spheroids or organoids; see the growing number of studies [289,290,291,292,293,294,295,296]) or animal models (e.g., syngenic, genetically engineered (GEMMs), PDXs (reviewed in [297,298])), are essential for studying UM and evaluating potential therapeutic interventions. Furthermore, the establishment of a robust zebrafish PDX (zf-PDX) platform for UM research offers a new horizon for efficient drug screening. This model, involving the engrafting and propagation of human tumor materials in zebrafish hosts, holds promise for personalized medicine applications [299]. In addition, the use of the chick embryo chorioallantoic membrane (CAM) model, already established for UM fundamental research [300], showed recently a potential for an effective and economical model for preclinical assessment, as well as for enhancing the selection of drug candidates and developing new effective treatment strategies for UM [301]. Interestingly, *BAP1*-deficient *Xenopus laevis* embryos were used previously for a drug screening and allowed to identify the HDAC4 inhibitor quisinostat as a candidate for the treatment of *BAP1*-mutant UMs [302]. PDXs might serve effectively as “avatars” in selecting the optimal personalized treatment for patients with the highest risk of relapse [303]. Finally, a recent study outlined an innovative method for creating personalized brachytherapy implants, combining 3D printing with PEEK polymer, biomedical μCT imaging for precise geometrical validation, and a novel “radioactivity painting” technique for dose modulation, offering a tailored fit for complex anatomical and tumor shapes, potentially enhancing the efficacy of radiotherapy treatments [304]. Future research should aim to develop more accurate and representative preclinical models, including tissue-engineered (reviewed in [5,305]) or microfluidic tumor models, bioprinted hydrogel phantoms, patient-derived organoids (PDOs) or patient-derived organotypic tumor spheroid (PDOTSs), as well as humanized and chimeric mice, that mimic the genetic, histologic, immunologic, and metastatic features of UM [306].

### 7.2. Limited Treatment Options for MUM

Once UM becomes metastatic, therapeutic options are limited, and treatment strategies are often extrapolated from CM successes (reviewed in [307]). As of now, Tebentafusp has been the sole therapy for MUM to show a higher OS benefit (73%) in adult with HLA-A*02:01 positive status, whereas Pembrolizumab demonstrated a 59% OS rate in similar patients [308]. The development of effective and universal therapies specifically tailored for MUM is crucial to improve patient outcomes.

### 7.3. Identification of Novel Therapeutic Targets

The identification of specific genetic alterations and signaling pathways in UM has opened-up opportunities for targeted therapies [309]. Future research should focus on further understanding these alterations and developing therapies that specifically target them.

### 7.4. Improved Diagnostic and Prognostic Tools

UM diagnosis and prognosis can be challenging. Advances in tumor biology and cytogenetic tests have the potential to improve diagnostic accuracy and provide valuable prognostic information [310]. Future research should aim to develop more precise and reliable diagnostic and prognostic tools as well as initiating treatment at earlier stages [311].

### 7.5. Exploration of Immunotherapeutic Approaches

Immunotherapies have shown promise in the treatment of various cancers. However, their efficacy in UM has improved but still remains limited (reviewed in [312]). Future research should focus on developing immunotherapeutic strategies that can specifically and effectively target UM TIME and improve patient outcomes.

### 7.6. Identification of Prognostic Biomarkers and Therapy Responses

Prognostic biomarkers play a crucial role in predicting disease progression and guiding treatment decisions. The identification of reliable prognostic biomarkers in UM is an ongoing research focus ([313], reviewed in [314]). Future research should aim to identify and validate prognostic biomarkers that can accurately predict patient outcomes and assess immunotherapy responses, such as molecular signature predictors or UM metastasis prediction score.

### 7.7. Intraoperative Imaging and Surgical Techniques

Intraoperative imaging techniques, such as intraoperative optical coherence tomography (OCT), can provide real-time visualization and guidance during surgical procedures [315]. Lately, the use of MRI contrast agents designed to target specific matrix proteins, abundant in the TME, such as collagen type-I and fibronectin, has enhanced the sensitivity of metastasis detection (e.g., liver) [316,317]. This approach has proven effective in precisely locating micro-metastases (0.5 mm) in vivo, highlighting its potential to significantly improve the non-invasive, early detection and staging of metastatic cancers [316,317]. Additionally, another study based on cancer cell metabolism developed new fluorescent probes using nitrogen-doped carbon dots that leverage the distinct energy metabolism patterns of tumor cells (including UM) to identify micron-sized tumor lesions and monitor tumor proliferation and metastasis in a murine ocular tumor model [318]. Lastly, elastography, a technique for assessing the mechanical characteristics of tissues within the body [319], has been exploited for intraocular tumors and provided a significant different measure of tissue elasticity between benign and malignant conditions [320]. Future research should focus on the development and refinement of intraoperative imaging techniques to improve surgical outcomes and minimize complications.

### 7.8. Evaluating the Relationship between the Microbiome and the Immunotherapy Response

The lack of response to immunotherapy in some patients may be influenced by their microbiome. While specific studies on UM are lacking, research on skin melanomas and other cancers suggests that modifying the gut microbiome through dietary changes or fecal transplants can enhance the effectiveness of these drugs [321,322]. This indicates a potential relationship between the gut microbiome and the response to immunotherapy, opening avenues for improving treatment outcomes through microbiome modulation.

### 7.9. Advancing the Development of Oncolytic Viruses

Specificity and cytolytic activity of the oncolytic viruses have shown insights into potentially new treatments in combination with immunotherapy (reviewed in [323]). This integrated approach could offer a more effective treatment regimen not only directly attacking the cancer cells but also by boosting the body immune response against the tumor.

### 7.10. Employing Artificial Intelligence (AI) in the Prognostic Analysis

In ocular oncology, classical machine learning remains prevalent due to scarce data (reviewed in [324]), but advanced deep learning techniques, like generative adversarial networks, are anticipated to overcome these limitations, especially in applications like prognostication in UM (reviewed in [325]).

## 8. Conclusions

In summary, UM stands as a distinct form of melanoma with a unique genetic and molecular landscape that drives its pathogenesis and progression. Differences in its mutational burden, cellular origin, and clinical behavior necessitate specialized approaches for treatment and management. The identification of early mutations in genes such as *GNAQ*/*11*, *BAP1* and *CYSLTR2*, among others, and the understanding of their roles in tumor growth and metastasis, have greatly enhanced our understanding of UM and provided potential targets for therapy. However, despite these advances, the prognosis for MUM remains poor, highlighting the urgent need for continued research and development of novel diagnostic/prognostic tools and therapeutic strategies. The clinical management of UM has benefited from molecular stratification, allowing for more personalized treatment and improved prognostication. Yet the challenge of managing MUM, particularly due to its predilection for liver metastasis, remains a significant hurdle. Innovations in liquid biopsy techniques, the discovery of novel biomarkers, and the advent of targeted therapies, like Tebentafusp, represent significant strides in the field, but the quest for more effective treatments continues. Emerging insights into UM TME/TIME and their influence on tumor behavior and treatment response are opening new avenues for immunotherapy, even as we grapple with the complexity of the immune response in UM. Advances in HDACi, MAPK pathway inhibitors, and the exploration of the Hippo/YAP pathway are providing new therapeutic possibilities. Furthermore, recent developments in drug delivery systems, such as nanoparticles and virus-like drug conjugates, alongside the application of selective internal radiation therapy, are promising strategies that may enhance the precision and efficacy of UM treatment. However, significant challenges persist, including the translation of preclinical findings to clinical success, the development of resistance to targeted therapies, and the need for better predictors of treatment response. As research continues to elucidate the intricate biology of UM, a multidisciplinary approach combining cutting-edge science with patient-centered care will be essential to improve outcomes for those affected by this challenging cancer. The shift in focus towards molecular biomarkers from metastases, as opposed to those of primary tumors, for personalizing therapy, underscores the need to prioritize access to molecular diagnostics for UM patients.

## Data Availability

The figures were created with BioRender.com (accessed on 15 March 2024) and cBioPortal (https://www.cbioportal.org/ accessed on 8 June 2024).
